# Cortical thinning in patients with REM sleep behavior disorder is associated with clinical progression

**DOI:** 10.1038/s41531-019-0079-3

**Published:** 2019-05-03

**Authors:** Joana B. Pereira, Daniel Weintraub, Lana Chahine, Dag Aarsland, Oskar Hansson, Eric Westman

**Affiliations:** 1 Division of Clinical Geriatrics, Department of Neurobiology, Care Sciences and Society, Karolinska Institutet, Stockholm, Sweden; 2 Department of Psychiatry, Perelman School of Medicine at the University of Pennsylvania, Philadelphia, PA USA; 3 PD Research, Education and Clinical Center, Philadelphia Veterans Affairs Medical Center, Philadelphia, PA USA; 4 Department of Neurology, Perelman School of Medicine at the University of Pennsylvania, Philadelphia, PA USA; 5 Department of Old Age Psychiatry, Institute of Psychiatry, Psychology & Neuroscience, King’s College London, London, England; 6 Centre for Age-Related Medicine, Stavanger University Hospital, Stavanger, Norway; 7 Clinical Memory Research Unit, Department of Clinical Sciences Malmö, Lund University, Lund, Sweden; 8 Memory Clinic, Skåne University Hospital, Malmö, Sweden

**Keywords:** Predictive markers, Neurodegenerative diseases

## Abstract

The aim of this study is to determine whether structural MRI measures are associated with clinical impairment and progression to a Lewy body disease in patients with idiopathic REM sleep behavior disorder (iRBD). Twenty-seven patients with iRBD in addition to patients with de novo PD and healthy controls were included from the Parkinson’s Progression Markers Initiative. Patients with iRBD were followed for up to 3 years. Clinical and MRI measures were compared across groups and the association between clinical features and structural MRI was assessed in iRBD patients. Cox regression analyses were applied to identify risk factors for progressing to a Lewy body disease in iRBD. Our results showed that, at baseline, iRBD patients showed parietal and occipital cortical thinning, compared to controls. They also showed worse motor and non-motor abilities, some of which correlated with motor, frontal or temporal cortical thinning. At follow-up, six (22%) iRBD patients were diagnosed with a Lewy body disorder. These patients showed cortical thinning in frontal, occipital and parietal areas compared to iRBD non-converters. Cortical thinning was a significant predictor for future development of a Lewy body disorder (HR: 0.784; 95% CI: 0.640–0.960; p = 0.02). We conclude that cortical thinning is associated with worse motor and non-motor abilities, and predicts conversion to a Lewy body disorder in iRBD, suggesting it could be used to select candidates for clinical trials to delay the onset of neurodegenerative disease.

## Introduction

It is becoming increasingly clear that many Lewy body disorders can be characterized by a prodromal phase during which nonmotor symptoms occur before a clinical diagnosis can be made.^1^ The strongest prodromal symptom associated with future risk of a Lewy body disease is idiopathic REM sleep behavior disorder (iRBD), a parasomnia associated with unpleasant dreams and vigorous behaviors during REM sleep.^2^ Several studies have shown that individuals with iRBD may be ideal candidates for neuroprotective trials since they have a near universal risk of developing a Lewy body disorder,^1,3^ namely Parkinson’s disease (PD), dementia with Lewy bodies (DLB), and multiple system atrophy (MSA). However, in order to plan such trials, it is essential to estimate when iRBD patients will convert to one of these diseases, preferably with a marker that could potentially be used across different studies.^4^

Cortical thickness is a sensitive marker of brain atrophy that measures the distance between the gray/white and pial surfaces with submillimeter accuracy.^5^ There is consistent evidence showing cortical thinning in frontal, temporal, occipital, or parietal areas in patients with PD, DLB, or MSA, in association with motor, nonmotor and cognitive abnormalities.^6–11^ However, it is still unclear whether these changes are already present in the prodromal stages of these diseases and whether they could be used to identify iRBD individuals with a higher risk of converting to one of them. Previous studies using structural magnetic resonance imaging (MRI) have shown cortical thinning in medial frontal, lateral frontal, postcentral, temporal, and occipital regions.^12–14^ However, these studies did not have information regarding conversion to a Lewy body disorder, which can occur at highly variable time intervals in iRBD.^15^ In addition, they did not compare the atrophy patterns in iRBD with those observed in PD.

To address these questions, we measured cortical thickness and subcortical volumes in patients with iRBD, patients with newly diagnosed PD and healthy controls. Specifically, our aims were to: (1) compare neuroanatomical and clinical markers between iRBD patients and the other groups; (2) explore the association between brain atrophy and clinical features in iRBD; and (3) test the ability of baseline atrophy patterns to predict which iRBD individuals will progress to a Lewy body disorder over the short term (i.e., up to a 3-year period). We hypothesized that cortical and subcortical changes would be detectable in iRBD patients at baseline and that these changes would correlate with clinical deficits and predict conversion to a Lewy body disorder.

## Results

### Clinical differences between groups

The clinical characteristics of our sample can be found in Table 1. In total, 27 patients with iRBD, 151 patients with newly diagnosed PD and 31 healthy controls were included.

**Table 1:** Clinical characteristics of iRBD patients, PD patients and controls

	iRBD (*n* = 27)	PD (*n* = 151)	CTR (*n* = 31)	iRBD vs. CTR (*p* value)	PD vs. CTR (*p* value)	iRBD vs. PD (*p* value)
Age (mean, SD)	68.9 (5.5)	60.6 (9.6)	58.5 (11.0)	**<0.001**	0.335	**<0.001**
Sex (M/F)	22/5	94/57	20/11	0.149	0.812	0.053
Education (mean, SD)	12.7 (5.2)	15.4 (2.9)	16.5 (3.1)	**0.006**	0.067	0.022
MDS-UPDRS III (mean, SD, range)	4.2 (3.6; 0–15)	20.5 (9.2; 0–51)	0.32 (0.9; 0–4)	**<0.001**	**<0.001**	**<0.001**
Hoehn and Yahr (mean, range)	0 (0–0)	1.6 (1–3)	0 (0–0)	1.000	**<0.001**	**<0.001**
UPSIT (mean, SD, range)	17.6 (6.2; 9–35)	21.9 (8.4; 1–39)	36.7 (1.6; 34–40)	**<0.001**	**<0.001**	0.436
RBDSQ (mean, SD, range)	9.3 (2.9; 1–13)	3.9 (2.6; 0–12)	2.1 (1.4; 0–4)	**<0.001**	**0.002**	**<0.001**
ESS (mean, SD, range)	8.4 (4.5; 0–20)	5.4 (3.2; 0–15)	4.8 (3.1; 0–12)	**0.007**	0.465	**0.002**
GDS (mean, SD, range)	6.0 (2.1; 3–11)	5.3 (1.5; 1–11)	5.2 (1.0; 2–7)	0.168	0.952	0.104
MoCA (mean, SD, range)	25.3 (4.5; 11–30)	27.3 (2.3; 19–30)	28.3 (1.2; 27–30)	0.279	0.224	0.587
Immediate recall (HVLT-R) (mean, SD, range)	20.7 (5.4; 9–33)	25.2 (5.4; 11–36)	26.7 (4.7; 16–35)	0.020	0.503	0.102
Delayed recall (HVLT-R) (mean, SD, range)	6.8 (3.0; 0–12)	8.6 (2.7; 0–12)	10.0 (1.9; 6–12)	0.210	0.057	0.908
Recognition (HVLT-R) (mean, SD, range)	10.4 (1.5; 7–12)	11.4 (1.0; 8–12)	11.7 (0.6; 10–12)	0.102	0.618	0.125
Benton Judgment Line Orientation (mean, SD, range)	11.5 (1.9; 8–15)	12.9 (2.0; 7–15)	13.3 (1.8; 9–15)	0.037	0.934	0.027
Letter and Number Sequencing (mean, SD, range)	8.6 (3.1; 4–17)	10.9 (2.9; 2–20)	11.9 (3.0; 8–20)	0.065	0.639	0.117
Semantic fluency (mean, SD, range)	44.3 (9.2; 27–65)	49.8 (12.1; 20–103)	55.2 (9.5; 39–74)	0.101	0.029	0.507
Symbol and Digit Modalities Test (mean, SD, range)	31.4 (9.3; 15–56)	41.6 (9.8; 7–70)	49.0 (11.5; 30–76)	**0.003**	**0.001**	0.037

Values correspond to means followed by standard deviation or standard deviation and range. Comparisons between groups were performed using *X*^2^, Mann–Whitney *U* tests, or ANOVA. Age and sex were included as covariates in the analyses of motor and nonmotor variables, whereas education was included as an additional covariate in the analyses of cognitive variables. Values in bold correspond to significant group differences after adjusting for multiple comparisons with false-discovery rate corrections (FDR) (*q* < 0.05)

Compared to controls, iRBD patients were significantly older (Cohen’s *d* = 1.196, *p* < 0.001), less educated (Cohen’s *d* = 0.888, *p* = 0.006) and had higher MDS-UPDRS III scores (Cohen’s *d* = 1.264, *p* < 0.001). In addition, they also had more daytime sleepiness (Cohen’s *d* = 0.773, ESS, *p* = 0.007) and performed significantly worse in cognitive tests measuring attention (Cohen’s *d* = 0.879, SDMT, *p* = 0.003). PD patients showed greater motor impairment (Cohen’s *d* = 3.142, MDS-UPDRS III, *p* < 0.001) and worse attention (Cohen’s *d* = 0.539, SDMT, *p* = 0.001) compared to controls, after FDR corrections.

When iRBD and PD patients were compared to each other, we found that iRBD patients were older (Cohen’s *d* = 1.023, *p* < 0.001) and more impaired in tests evaluating RBD (Cohen’s *d* = 0.319, RBDSQ, *p* < 0.001) and sleepiness (Cohen’s *d* = 0.668, ESS, *p* = 0.002), after FDR corrections. On the other hand, as expected, PD patients showed greater motor impairment (Cohen’s *d* = 2.419, MDS-UPDRS III, *p* < 0.001) compared to iRBD.

### MRI differences between groups

**Fig. 1:**
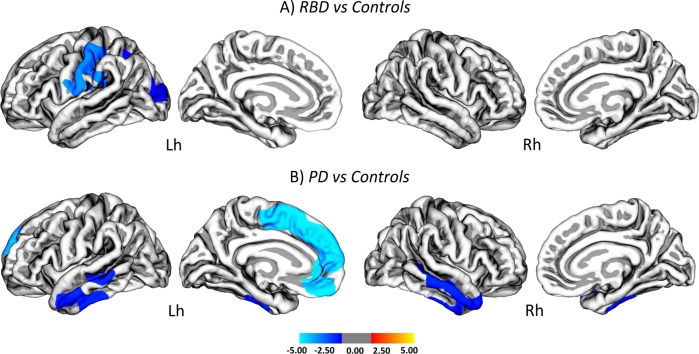
Cortical thinning in patients with iRBD and patients with newly diagnosed PD compared to controls. Vertex-wise comparisons of cortical thickness between: **a** controls and patients with idiopathic REM sleep behavior disorder (iRBD); and **b** CTR and patients at early stages of Parkinson’s disease (PD). The color scale bar shows the logarithmic scale of *p* values (−log_10_). All results were adjusted for multiple comparisons (cluster-wise threshold *p* < 0.05 with Monte Carlo simulations) and corrected for age, sex and education. Lh left hemisphere, Rh right hemisphere

In patients with iRBD, cortical thinning was found in the left lateral occipital (Cohen’s *d* = 0.586; *p* = 0.03) and postcentral (Cohen’s *d* = 0.786; *p* = 0.002) gyri, which extended to left inferior parietal and supramarginal areas (Fig. 1, Table 2) compared to controls.

**Table 2:** Regions that showed cortical thinning in iRBD patients and PD patients compared to controls

Cortical area	Effect size (Cohen’s *d*)	Cluster size (mm^3^)	Cluster-wise *p* value	Talairach coordinates		
				*x*	*y*	*z*
*iRBD vs. controls*						
Lh lateral occipital G	0.586	1999.09	0.03370	−29.5	−84.9	16.2
Lh postcentral G	0.786	3064.10	0.00200	−60.1	−8.8	19.8
*PD vs. controls*						
Lh inferior temporal G	0.552	2971.71	0.00410	−53.5	−26.1	−24.7
Lh superior frontal G	0.407	4625.03	0.00020	−7.6	−4.9	51.5
Rh inferior temporal G	0.451	2815.45	0.00430	47.5	−18.6	−26.3

*Lh* left hemisphere, *Rh* right hemisphere, *G* gyrus. All results were corrected for multiple comparisons using a cluster-wise threshold of *p* < 0.05 with Monte Carlo simulations. In addition they were also adjusted for age, sex, and education

In patients with PD, there was significant cortical thinning in the bilateral inferior temporal (left: Cohen’s *d* = 0.552; *p* = 0.004; right: Cohen’s *d* = 0.451; *p* = 0.004) and left superior frontal (Cohen’s *d* = 0.407; *p* < 0.001) gyri compared to controls (Fig. 1, Table 2).

There were no significant differences in subcortical volumes between groups (Supplementary Table 1) or differences in cortical thickness between iRBD and PD patients.

### MRI measures correlate with clinical impairment in iRBD

**Fig. 2:**
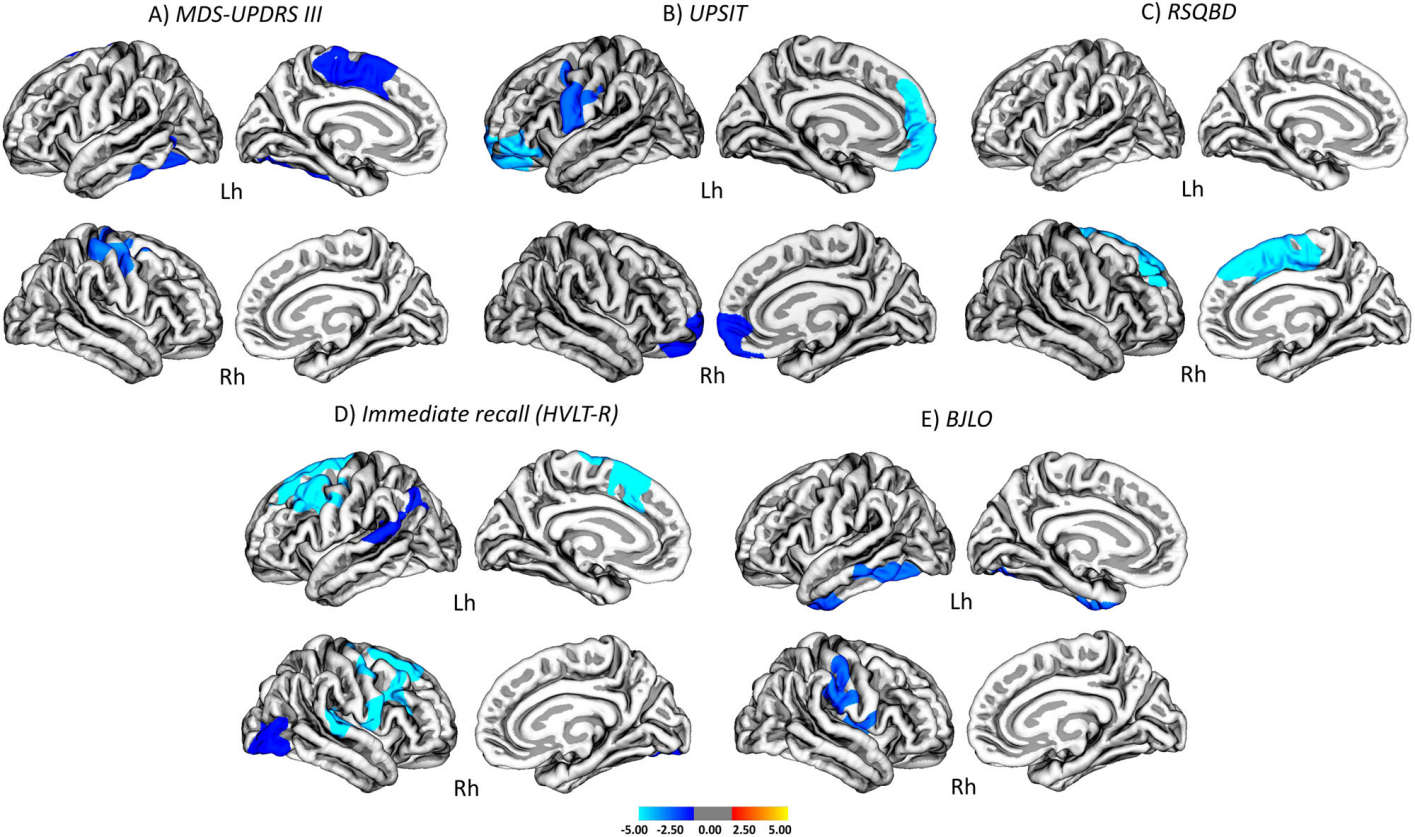
Associations between cortical thinning and motor, nonmotor and cognitive deficits in patients with iRBD. Significant vertex-wise correlation between cortical thinning and **a** Movement Disorders Society Unified Parkinson’s Disease Rating Scale (MDS-UPDRS) III motor scores; **b** University of Pennsylvania Smell Identification Test scores; **c** Rapid Eye Movement Behavior Disorder Sleepiness Questionnaire (RBDSQ) scores; **d** Immediate recall scores of the Hopkins Verbal Learning Test—Revised (HVLT-R); and **e** Benton Judgment of Line Orientation (BJLO) test visuospatial scores. The color scale bar shows the logarithmic scale of *p* values (−log_10_). All results were adjusted for multiple comparisons (cluster-wise threshold *p* < 0.05 with Monte Carlo simulations) and corrected for age, sex (all correlations) in addition to education (correlations with cognition). Lh left hemisphere, Rh right hemisphere

There were several significant correlations between cortical thickness and clinical measures in iRBD (Fig. 2, Supplementary Table 2). Increasing motor disease severity (MDS-UPDRS III) was associated with cortical thinning in the left superior frontal (*r* = −0.584; *p* = 0.021), left fusiform (*r* = −0.489; *p* = 0.011) and right precentral gyri (*r* = −0.447; *p* = 0.002). Moreover, worse olfaction (UPSIT) correlated with left medial orbitofrontal (*r* = 0.478; *p* < 0.001), left precentral (*r* = 0.468; *p* = 0.003), and right medial orbitofrontal (*r* = 0.468; *p* = 0.02) thinning, whereas RBD (RBDSQ) correlated with right superior frontal (*r* = 0.466; *p* < 0.001) thinning.

Regarding cognitive tests, we found that worse memory performance (HVLT-R Immediate recall) correlated with cortical thinning in the left superior temporal (*r* = 0.453; *p* = 0.02), left caudal middle frontal (*r* = 0.419; *p* < 0.001), right superior frontal (*r* = 0.447; *p* < 0.001) and right lateral occipital (*r* = 0.449; *p* = 0.03) gyri; whereas visuospatial impairment (BJLO) correlated with left fusiform (*r* = 0.400; *p* = 0.001) and right supramarginal (*r* = 0.390; *p* = 0.004) thinning. No significant correlations were found with subcortical regions.

### Differences between converters and nonconverters

All 27 iRBD patients were clinically assessed after a few years. As a result of this clinical assessment, six iRBD patients (22.2%) were clinically diagnosed with a Lewy body disorder: three patients were diagnosed with PD, one patient with DLB, one patient with MSA, and one patient with nonspecific parkinsonism. The remaining 21 iRBD patients did not convert to another disorder at follow-up. The clinical characteristics for both converters and nonconverters can be found in Table 3. The last available follow-up for the converters was 2.5 years (3 patients), 3 years (1 patient), and 3.5 years (2 patients), whereas the last available follow-up for the nonconverters was 1.5 years (1 patient), 2 years (4 patients), 2.5 years (2 patients), 3 years (10 patients), and 3.5 years (4 patients) (Table 3).

**Table 3:** Clinical characteristics of iRBD patients that converted to as Lewy body disorder and nonconverters

	Converters (*n* = 6)	Non-converters (*n* = 21)	Converters vs. nonconverters (*p* value)
Age (mean, SD)	67.8 (4.1)	69.2 (5.9)	0.593
Sex (M/F)	4/2	18/3	0.289
Education (mean, SD)	11.8 (5.0)	13.0 (5.3)	0.649
Years between baseline and follow-up (mean, SD, range)	2.9 (0.5, 2.5–3.5)	2.8 (0.5, 2.0–3.5)	0.798
Years to PD conversion (mean, SD, range)	2.3 (0.3, 2.0–2.5)	–	–
Last available follow-up (number of patients assessed after 1.5/2/2.5/3/3.5 years)	0/0/3/1/2	1/4/2/10/4	0.798
MDS-UPDRS III Baseline (mean, SD, range)	5.8 (4.8; 2–15)	3.7 (3.2; 0–12)	0.409
Follow-up (mean, SD, range)	22.8 (14.8; 9–49)	4.4 (3.9; 0–14)	**<0.001**
UPSIT baseline (mean, SD, range)	16 (9.6; 9–35)	18.1 (5.1; 9–31)	0.440
RBDSQ baseline (mean, SD, range)	11.0 (1.8; 8–13)	8.9 (3.1; 1–12)	0.151
Follow-up (mean, SD, range)	8.5 (4.7; 0–13)	7.6 (3.9; 0–13)	0.270
ESS baseline (mean, SD, range)	11.0 (5.9; 6–20)	7.7 (3.9; 0–16)	0.255
Follow-up (mean, SD, range)	12.0 (5.1; 6–20)	5.6 (3.5; 1–13)	0.040
GDS baseline (mean, SD, range)	7.5 (2.4; 5–11)	5.6 (1.8; 3–9)	0.205
Follow-up (mean, SD, range)	7.0 (0.9; 6–8)	5.5 (1.6; 2–8)	0.232
MoCA baseline (mean, SD, range)	23.2 (6.1; 11–27)	25.9 (4.0; 14–30)	0.422
Follow-up (mean, SD, range)	22.3 (4.6; 14–27)	25.6 (3.2; 18–30)	0.223
Immediate recall (HVLT-R) baseline (mean, SD, range)	16.3 (4.3; 9–22)	21.9 (5.1; 13–33)	0.031
Follow-up (mean, SD, range)	16.0 (4.9; 11–22)	22.3 (6.7; 4–34)	0.028
Delayed recall (HVLT-R) baseline (mean, SD, range)	5.0 (3.8; 0–11)	7.3 (2.6; 3–12)	0.058
Follow-up (mean, SD, range)	5.7 (4.0; 1–12)	7.5 (2.4; 1–11)	0.100
Recognition (HVLT-R) baseline (mean, SD, range)	9.3 (1.5; 8–12)	10.7 (1.3; 7–12)	0.041
Follow-up (mean, SD, range)	8.7 (3.3; 3–12)	10.7 (1.4; 7–12)	0.047
Benton judgment line orientation baseline (mean, SD, range)	10.2 (2.4; 8–14)	11.8 (1.7; 8–15)	0.083
Follow-up (mean, SD, range)	11.6 (2.1; 9–14)	10.4 (2.4; 5–15)	0.404
Letter and number sequencing baseline (mean, SD, range)	7.2 (2.1; 4–9)	9.0 (3.3; 4–17)	0.699
Follow-up (mean, SD, range)	6.2 (3.3; 3–11)	8.2 (2.9; 4–15)	0.596
Semantic fluency baseline (mean, SD, range)	41.5 (8.6; 35–56)	45.1 (9.4; 27–65)	0.429
Follow-up (mean, SD, range)	41.0 (7.7; 34–53)	44.3 (10.8; 26–64)	0.465
Symbol and digit modalities test baseline (mean, SD, range)	32.0 (6.1; 24–41)	31.2 (10.0; 15–56)	0.798
Follow-up (mean, SD, range)	32.2 (7.3; 23–40)	29.5 (11.9; 0–54)	0.732

Values correspond to means followed by standard deviation or standard deviation and range. Comparisons between groups were performed using *X*^2^, Mann–Whitney *U* tests, or ANOVA. Age and sex were included as covariates in the analyses of motor and nonmotor variables, whereas education was included as an additional covariate in the analyses of cognitive variables. Values in bold correspond to significant group differences after adjusting for multiple comparisons with false-discovery rate corrections (FDR) (*q* < 0.05)

At baseline, there were no significant differences in clinical variables between patients that converted to a Lewy body disorder and those that remained disease free, after adjusting for multiple comparisons. After approximately 3 years, these patients showed greater motor disease severity (MDS-UPDRS III, *p* < 0.001) in line with their conversion to a Lewy body disorder, compared to nonconverters.

**Fig. 3:**
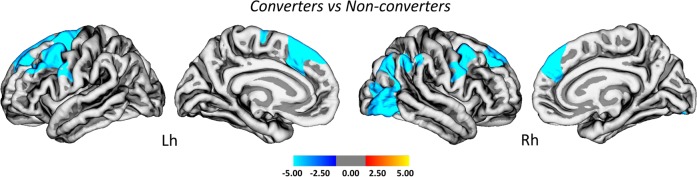
Cortical thinning in iRBD patients that converted to a Lewy body disorder compared to non-converters. Vertex-wise comparisons of cortical thickness between patients with idiopathic REM sleep behavior disorder (iRBD) that progressed to a Lewy body disorder at follow-up (converters) compared to iRBD patients that remained disease free (nonconverters). The color scale bar shows the logarithmic scale of *p* values (−log_10_). All results were adjusted for multiple comparisons (cluster-wise threshold *p* < 0.05 with Monte Carlo simulations) and corrected for age, sex, education, baseline MDS-UPDRS III motor scores and time interval between baseline and last follow-up assessment. Lh left hemisphere, Rh right hemisphere

The analyses of cortical thickness using the baseline MRI images revealed widespread thinning in the left superior frontal (Cohen’s *d* = 1.191, *p* < 0.001), right precentral (Cohen’s *d* = 1.128, *p* < 0.001) and right lateral occipital gyri (Cohen’s *d* = 1.130, *p* < 0.001) in converters compared to nonconverters (Fig. 3, Supplementary Table 3). No differences were found in subcortical regions between these groups.

The comparisons between healthy controls and converters, and healthy controls and non-converters have been included in Supplementary Fig. 1 and Supplementary Table 4. Compared to controls, converters showed even more widespread cortical thinning in similar frontal, precentral and occipital areas. Although no significant differences were found in nonconverters compared to controls after correcting for multiple comparisons, at an uncorrected level there was cortical thinning in brain regions that were similar to the ones observed in the whole iRBD group (Fig. 1). No significant differences were found in subcortical volumes between groups, after FDR corrections (Supplementary Table 5).

### Cortical thinning as a risk factor for developing a Lewy body disorder

We built a Cox univariate model using the mean cortical thickness extracted from the brain vertices showing significant group differences in Fig. 3 to assess whether it could predict conversion to a Lewy body disorder. The results showed that the mean cortical thickness significantly predicted conversion to a Lewy body disease (HR = 0.784; 95% CI: 0.640–0.960; *p* = 0.019), with a sensitivity of 95.2%, specificity of 100% and an AUC of 0.984 (CI 95%: 0.945–1.000; *p* < 0.001).

### Influence of probable RBD in PD patients

Although PD patients did not undergo polysomnography to confirm the presence of RBD, to assess the potential influence of RBD symptoms in the clinical and MRI profiles of these patients, we used the scores from the rapid eye movement behavior disorder (RBD) Questionnaire—RBDSQ (cut-off ≥ 5) to divide them into two groups with probable presence (*n* = 56) or absence (*n* = 94) of RBD, similarly to a previous study.^16^

Compared to controls, PD patients without probable RBD had greater motor impairment (Cohen’s *d* = 3.074, *p* < 0.001) and olfactory dysfunction (Cohen’s *d* = 2.107, <0.001), whereas PD patients with probable RBD, in addition to motor and olfactory deficits, presented greater sleepiness (Cohen’s *d* = 0.210, *p* = 0.001), memory impairment (delayed recall, Cohen’s *d* = 0.741, *p* = 0.001) and semantic fluency impairment (Cohen’s *d* = 0.910, *p* < 0.001) (Supplementary Table 6).

Compared to iRBD patients, both PD patients with and without probable RBD were more educated, had greater motor impairment, less sleepiness and more RBD symptoms (*p* range: 0.016–<0.001). In addition, PD patients without RBD were less impaired in memory (Cohen’s *d* = 0.462, recognition, *p* = 0.008) and visuospatial (Cohen’s *d* = 0.646, BJLO, *p* = 0.005) tests, whereas PD patients with probable RBD were less impaired in semantic fluency (Cohen’s *d* = 0.643, *p* = 0.011) compared to iRBD.

The cortical thickness analyses showed that PD patients without probable RBD had cortical thinning only in the left superior frontal gyrus (Cohen’s *d* = 0.407, *p* = 0.002), whereas PD patients with RBD had more widespread cortical thinning in bilateral superior frontal (left: Cohen’s *d* = 0.737, *p* < 0.001; right: Cohen’s *d* = 0.508, *p* < 0.001), bilateral inferior temporal (left: Cohen’s *d* = 0.540, *p* = 0.003; right: Cohen’s *d* = 0.548, *p* = 0.0013) and left rostral middle frontal (left: Cohen’s *d* = 0.508, *p* < 0.001) regions (Supplementary Fig. 2; Supplementary Table 7), compared to controls. These results suggest that the presence of RBD symptoms in PD is associated with greater cortical changes. There were no significant differences in cortical thickness between the PD groups and iRBD patients.

## Discussion

In this longitudinal study we explored the potential of structural neuroimaging to identify brain abnormalities in individuals with iRBD enriched for incipient parkinsonism, their relationship with clinical impairment and value as risk markers to develop an imminent neurodegenerative disease. Our main findings showed a pattern of cortical thinning in iRBD compared with controls, which was different from the one observed in PD patients. There were also several significant correlations between cortical thickness and motor, nonmotor and cognitive measures in iRBD. Finally, we found that cortical thinning in frontal, occipital and parietal areas predicted a substantially increased risk of progression to a clinically defined Lewy body disorder in iRBD after a relatively short period of 3 years. These findings suggest that cortical thickness could potentially be used to identify iRBD individuals who will convert faster to a neurodegenerative disease.

The prodromal stage of many Lewy body disorders is a period where motor, nonmotor and cognitive clinical manifestations occur but motor features are too subtle to allow a formal diagnosis of disease.^1^ Our findings are in line with this as we found worse motor, sleep, and attention symptoms or functions in iRBD patients. These clinical changes were accompanied by cortical thinning in left motor, parietal, occipital areas. This asymmetric pattern of atrophy was in line with the cortical thinning pattern observed in newly diagnosed PD patients in the current study, which was also more prominent in the left hemisphere. In addition, this atrophy pattern partially overlapped with a metabolic brain network that has previously shown to be implicated in iRBD.^17^ This network has been described in multiple studies^18^ and includes lateral occipital and parietal regions, among other areas. These regions have shown to present hypometabolism in iRBD and they also overlap with the areas of a metabolic network that is affected in PD.^18,19^ Together with our findings, these results suggest that occipital and parietal areas might be especially vulnerable to iRBD and could potentially represent an early manifestation of preclinical PD.

Despite the low number of phenoconverters in our study, we found that the most frequent disorder to which iRBD patients converted to was PD. This finding is in line with previous evidence showing that 50% of iRBD cases convert to PD within 5 years.^1^ In addition, we did not find any significant differences in cortical thickness between iRBD and PD patients, despite the fact that their baseline atrophy patterns were quite different with respect to controls. The absence of significant differences between these two patient groups could be due to the potentially high prevalence of future PD converters in our iRBD group. It is possible that there is already ongoing subtle atrophy in iRBD in similar areas as the ones affected in PD, although this effect is not strong enough to be detected at baseline and after controlling for multiple comparisons.

The presence of RBD symptoms has been associated with a malignant PD subtype characterized by rapid progression in cognitive, motor and nonmotor symptoms over time.^20,21^ Our results agree well with these findings as we found that PD patients with probable RBD presented greater memory and executive impairment in addition to more widespread cortical thinning than PD patients without RBD. This suggests that the presence of RBD symptoms may be responsible for a worse prognosis in PD and greater cortical changes. However, future studies are needed to replicate these findings in PD patients with confirmed RBD based on polysomnography results, which unfortunately was not available for the PD patients included in the current study.

In addition, we found that almost all clinical tests that were impaired in iRBD were associated with cortical thinning in relevant brain areas. For instance, motor impairment correlated with thinning in motor areas such as the precentral gyrus and olfaction correlated with thinning in the medial orbitofrontal gyrus, which is adjacent to the olfactory bulb.^22–24^ Regarding cognition, we found that visuospatial functions correlated with right parietal regions and other areas that are important for visual perception,^24^ whereas memory correlated with left temporal regions, which are important for memory consolidation.^24^ Together, these findings suggest that cortical thinning might contribute to some of the clinical deficits observed in iRBD.

To our knowledge, the current study is the first in assessing the value of a structural neuroimaging biomarker in predicting short-term progression to a parkinsonian syndrome in patients with iRBD. Our findings suggest that cortical thinning in frontal, occipital and parietal areas is a significant predictor for early development of a Lewy body disorder. Due to the high variability in time intervals between iRBD diagnosis and phenoconversion, it is important to find biomarkers that are able to identify iRBD patients at high risk for early conversion into clinically defined synucleinopathies.^15^ Our findings suggest that cortical thickness could be one of these biomarkers.

Some limitations should be recognized in this study such as the potential bias of including very healthy controls in the Parkinson’s Progression Markers Initiative (PPMI) cohort with MoCA scores ≥27, and the fact that only 31 of these controls did not present RBD (≥5 on RBDSQ) or olfactory dysfunction. In addition, the sample size of the converters group was very small so our findings should be interpreted with caution and replicated in larger, separate samples of iRBD patients that convert either to PD, DLB, or MSA, which may have different brain atrophy patterns.

Another limitation is the fact that patients with iRBD were older, less educated and more cognitively impaired compared to controls. The presence of cognitive deficits has been associated with RBD symptoms in PD, suggesting that cognition and sleep disturbances are not independent from each other.^21^ Regarding age and education, we included these variables as covariates of no interest in the comparisons between controls and iRBD patients. In addition, we also observed cortical thinning in iRBD converters compared to nonconverters, who did not differ in age or education. Hence, our MRI findings are most likely not related to age or education differences between the groups. One additional limitation is the fact that one of the iRBD converters was diagnosed with non-specific parkinsonism at follow-up; hence, we do not yet know which specific disease this patient had. Finally, the last available follow-up varied between iRBD patients, with some having a longitudinal assessment after 1.5 or 2 years and others having an assessment after 3 and 3.5 years. Although there were no significant differences in the last available follow-up between converters and non-converters, it would have better that all patients had been followed for the same number of years. Future studies with more homogeneous longitudinal evaluations are needed to assess whether this variable has any effect on the findings.

In summary, we found that cortical thinning is a useful marker to detect iRBD patients with an increased risk of short-term conversion to a Lewy body disorder, suggesting it could potentially be used in future neuroprotective trials aimed at preventing or delaying the onset of motor disease.

## Methods

### Participants

Data used in this article were obtained from the PPMI database (www.ppmi-info.org/data),^25^ accessed on May 20, 2017. For up-to-date information on the study, visit www.ppmi-info.org. For the purposes of this study, only iRBD patients, PD patients and healthy controls with a T1-weighted scan, that passed quality control before and after image preprocessing, were included.

Healthy controls were required not to have significant neurological dysfunctions, first-degree family members with PD, or cognitive impairment (Montreal Cognitive Assessment^26^ (MoCA; score ≥27). In addition, in order to maximize the likelihood of being free from a subclinical neurodegenerative disorder, controls who screened positive for RBD (≥5 on the Rapid Eye Movement Behavior Disorder (RBD) Questionnaire—RBDSQ)^27^ or had significant olfactory dysfunction for their age and sex^28^ were excluded from this study.

iRBD patients were required to have a diagnosis of iRBD based on clinical history and polysomnography results. To enrich this cohort with individuals presumed to have an incipient parkinsonian syndrome, most iRBD patients from PPMI had a dopamine transporter imaging (DaTscan) deficit. At the time of scanning, all iRBD patients were free of neurological diseases and did not present with significant parkinsonism. iRBD patients were followed for approximately 3 years. At each visit, a neurological examination was performed to apply clinical criteria for the diagnosis of neurodegenerative disorders, including PD, DLB, and MSA.

PD patients were required at baseline to meet standard diagnostic criteria for PD, have been diagnosed within 2 years, be untreated for PD and present a significant DaTscan deficit.

### Clinical evaluations

All subjects underwent a comprehensive assessment of motor, nonmotor and cognitive functions. Motor severity and disease stage were assessed using the Movement Disorders Society Unified Parkinson’s Disease Rating Scale (MDS-UPDRS) part III scores^29^ and the Hoehn & Yahr scale.^30^ Nonmotor functions were assessed using the University of Pennsylvania Smell Identification Test (UPSIT)^28^ (olfaction), the Epworth Sleepiness Scale (ESS)^31^ (daytime sleepiness), the RBDSQ^27^ (RBD) and the Geriatric Depression Scale-15 (GDS-15)^32^ (depression). Cognitive assessments included the MoCA^26^ (global cognition); the immediate, delayed and recognition recall scores of the Hopkins Verbal Learning Test–Revised (HVLT-R)^33^ (memory); the Benton Judgment of Line Orientation test (visuospatial functions)^34^; the Letter Number Sequencing (LNS)^35^ test and semantic fluency tests (executive functions); and the Symbol Digit Modalities Test^36^ (attention).

### Ethical approval

Each participating PPMI site received approval from an ethical standards committee on human experimentation before study initiation, and obtained written informed consent for research from all individuals participating in the study.

### MRI acquisition and preprocessing

All subjects were scanned on a 3T Siemens Tim Trio scanner using a high-resolution T1-weighted scan, acquired with a magnetization-prepared rapid acquisition gradient echo sequence (176 slices; repetition time = 1900–2300 ms; echo time = 2.27–2.98 ms; inversion time = 900 ms; flip angle = 9°; voxel size = 1 mm^3^ isotropic). T1-weighted images were preprocessed using FreeSurfer (version 6.0; http://freesurfer.net/) as published elsewhere.^37,38^ For every subject, a cortical surface model was generated, providing a measure of cortical thickness at each vertex. The final cortical maps were smoothed using a 15-mm full width at half maximum kernel. The volumes of subcortical gray matter structures (hippocampus, amygdala, thalamus, caudate, putamen, pallidum, accumbens) were also obtained from Freesurfer^39^ in addition to the estimated total intracranial volume (TIV).^40^

### Statistical analyses

#### Clinical group comparisons at baseline

Differences between groups in demographic and clinical variables were analyzed using chi-squared tests (*X*^2^), Mann–Whitney *U* tests or analysis of variance (ANOVA) in SPSS 24.0 (IBM Corp., Armonk, NY), while controlling for age and sex (motor and nonmotor variables) and additionally education (cognitive variables). To adjust the results for multiple comparisons, false-discovery rate (FDR) corrections^41^ were applied at *q* > 0.05.

#### MRI group comparisons at baseline

To assess cortical thickness differences between groups, a general linear model was estimated at each vertex using FreeSurfer. In this general linear model, cortical thickness was included as the dependent variable; group as a factor; and age, sex and education as nuisance variables. To adjust the results for multiple comparisons, Monte Carlo simulations with 10,000 iterations were applied (cluster-wise threshold *p* < 0.05). We calculated the Cohen’s effect size for all significant group comparisons.

Differences between groups in subcortical gray matter volumes were assessed using an ANOVA in SPSS, while controlling for the previous covariates in addition to TIV. To adjust the subcortical results for multiple comparisons, FDR corrections (*q* < 0.05) were applied.

#### Association between clinical impairment and MRI in iRBD

To assess the relationship between cortical thickness and the clinical tests that showed deficits in iRBD patients compared to controls, we estimated a general linear model that included cortical thickness as the dependent variable; clinical test scores as predictors; and age, sex, and education as nuisance variables. The correlation coefficients of the significant associations between thickness and clinical variables were calculated.

Partial correlation analyses were also carried out between subcortical volumes and clinical impairment, while adjusting for the previous covariates, TIV and FDR corrections.

#### Follow-up group comparisons

Patients with iRBD were followed up for approximately 3 years (mean = 2.8, range: 2.0–3.5). The diagnosis and clinical test scores were recorded at the last visit for all patients. We divided iRBD patients into two groups: (i) those who converted to a Lewy body disorder (converters) and (ii) those who did not convert to any neurodegenerative disorder (nonconverters). Differences between these groups in baseline clinical and imaging measures were assessed using an ANOVA or general linear models, similarly to previous analyses. In addition, to assess differences in all clinical variables across time we used repeated-measures ANOVAs, including clinical test scores at baseline and follow-up as dependent variables and group as a factor. All analyses were adjusted for age, sex, education, baseline MDS-UPDRS III scores, and FDR corrections.

#### Risk factor analysis

To assess the predictive ability of baseline brain atrophy for conversion to a Lewy body disorder in patients with iRBD, Cox regression analyses were performed using incident Lewy body disorder over 3 years as the outcome. We calculated hazard ratios, while adjusting for age, sex and education. Finally, we generated receiver operating characteristic curves for the significant predictors and calculated the area under the curve, sensitivity, and specificity.

### Reporting Summary

Further information on experimental design is available in the Nature Research Reporting Summary linked to this article.

## Supplementary information

Supplementary material

Reporting summary

## Data Availability

The data that support the findings of this study are available from the corresponding authors upon reasonable request.

## Supplementary information

**Supplementary information** accompanies the paper on the *npj Parkinson’s Disease* website (10.1038/s41531-019-0079-3).

## Code availability

As previously mentioned in the MRI subsection, all T1-weighted images were preprocessed using FreeSurfer (version 6.0).
