# Supplementary material for: Cortical thinning in patients with REM sleep behavior disorder is associated with clinical progression
Source: NPJ Parkinsons Dis. 2019 May 3;5:7. doi: 10.1038/s41531-019-0079-3 (PMC6499806; doi:10.1038/s41531-019-0079-3)
Supplement: Supplementary file 1 — Supplementary material [file 41531_2019_79_MOESM1_ESM.pdf]

Supplementary Figure 1. Cortical thinning in iRBD patients that converted to a Lewy body disorder and non-converters compared to controls

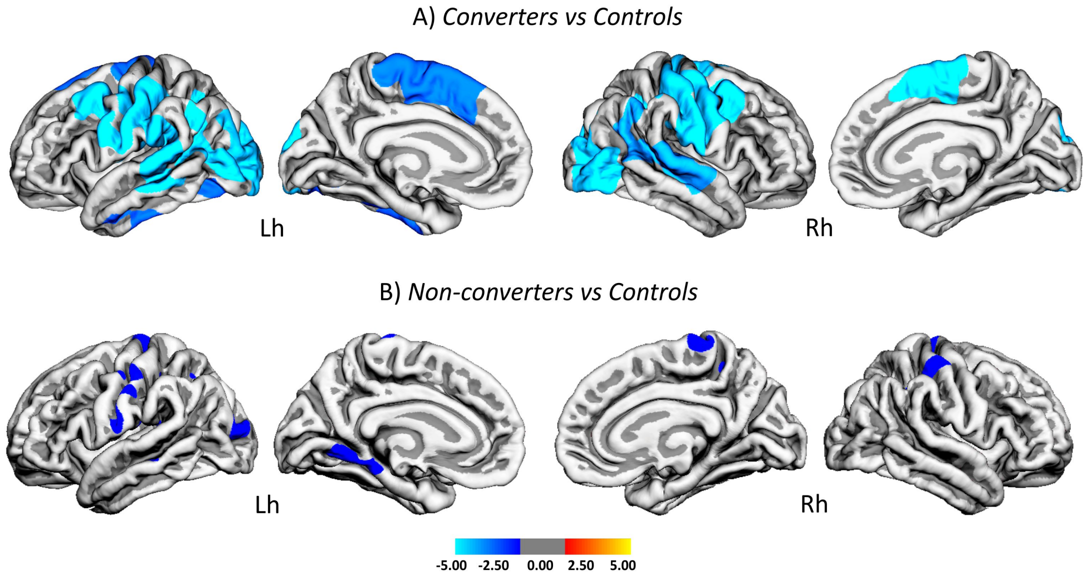

Vertex-wise comparisons of cortical thickness between: (A) controls and converters adjusted for multiple comparisons (cluster-wise threshold $P < .05$ with Monte Carlo simulations), and B) controls and non-converters unadjusted for multiple comparisons. The color scale bar shows the logarithmic scale of p values ($-\log_{10}$). The results were corrected for age, sex and education. Lh, left hemisphere; Rh, right hemisphere.

Supplementary Figure 2. Cortical thinning in PD patients with and without probable RBD compared to controls

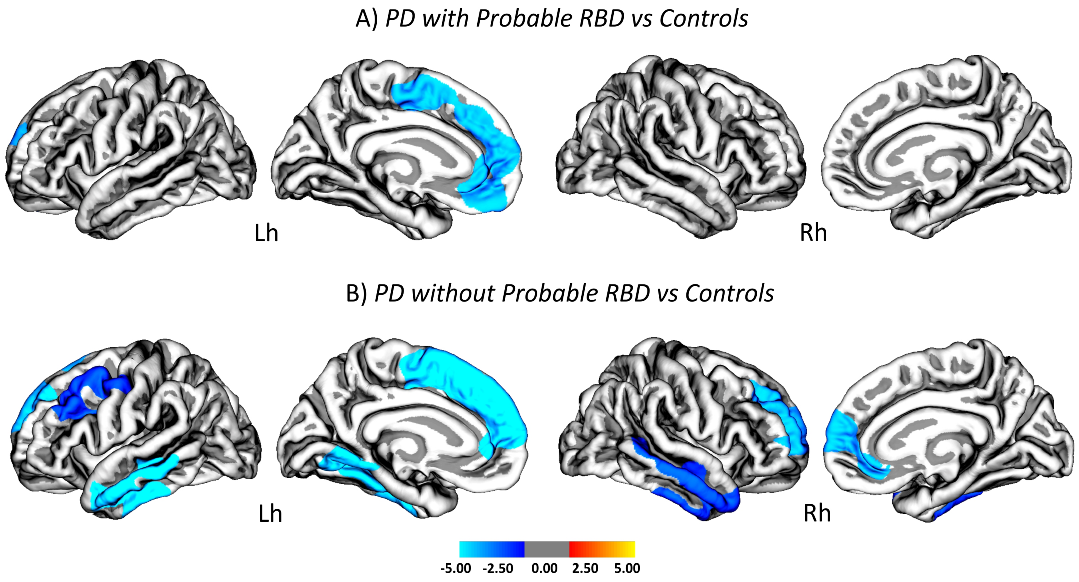

Vertex-wise comparisons of cortical thickness between: (A) controls and PD patients withprobable RBD adjusted for multiple comparisons (cluster-wise threshold P < .05 with Monte Carlo simulations), and B) controls and PD patients without probable RBD adjusted for multiple comparisons. The color scale bar shows the logarithmic scale of p values ($-\log_{10}$). The results were corrected for age, sex and education. Lh, left hemisphere; Rh, right hemisphere.

Supplementary Table 1. Subcortical volumes in iRBD patients, PD patients and controls

Subcortical area	iRBD	PD	CTR	iRBD vs CTR (p value)	PD vs CTR (p value)	iRBD vs PD (p value)
**Lh Thalamus**	6838.0 (806.7)	7246.3 (796.2)	7060.1 (864.4)	0.549	0.717	0.738
**Lh Caudate**	3387.8 (727.4)	3450.6 (466.9)	3376.3 (404.7)	0.518	0.795	0.561
**Lh Putamen**	4564.1 (850.0)	4706.4 (683.3)	4658.1 (560.3)	0.440	0.480	0.956
**Lh Pallidum**	1921.4 (287.8)	2076.4 (274.9)	1953.0 (222.4)	0.364	0.194	0.057
**Lh Accumbens**	414.4 (96.3)	478.2 (112.0)	446.1 (72.9)	0.622	0.286	0.176
**Lh Hippocampus**	3769.5 (414.2)	4055.0 (464.0)	3904.3 (371.4)	0.954	0.407	0.058
**Lh Amygdala**	1481.3 (202.9)	1630.8 (229.6)	1609.4 (141.4)	0.046	0.529	0.030
**Rh Thalamus**	7003.2 (953.6)	7241.5 (788.6)	7119.3 (954.1)	0.994	0.429	0.513
**Rh Caudate**	3499.4 (561.6)	3561.6 (481.0)	3401.3 (392.9)	0.932	0.576	0.937
**Rh Putamen**	4387.5 (749.8)	4672.8 (599.5)	4549.4 (556.8)	0.713	0.869	0.341
**Rh Pallidum**	1886.0 (282.4)	1965.6 (261.5)	1834.6 (202.2)	0.407	0.099	0.105
**Rh Accumbens**	447.9 (84.3)	513.2 (109.4)	477.2 (58.4)	0.197	0.251	0.160
**Rh Hippocampus**	3878.8 (408.3)	4160.4 (476.9)	4063.7 (409.5)	0.537	0.907	0.156
**Rh Amygdala**	1672.1 (239.6)	1739.1 (237.4)	1718.7 (137.6)	0.658	0.490	0.766

Lh, left hemisphere; Rh, right hemisphere; G, gyrus. Comparisons between groups were performed using ANOVA. All results were corrected for age, sex, education, TIV, and adjusted for multiple comparisons using FDR (q > 0.05).

Supplementary Table 2. Regions that showed a significant association between cortical thickness and clinical impairment in iRBD patients

**MDS-UPDRS III**						
**Cortical area**	**Correlation Coefficient**	**Cluster size (mm3)**	**Cluster-wise p-value**	**Talairach coordinates**		
				**x**	**y**	**z**
Lh Superior Frontal G	-0.489	2074.86	0.02070	-8.0	-9.6	51.4
Lh Fusiform G	-0.584	2305.99	0.01090	-41.7	-66.3	-11.8
Rh Precentral G	-0.447	2923.68	0.00190	33.4	-12.8	36.9
**UPSIT**						
**Cortical area**	**Correlation Coefficient**	**Cluster size (mm3)**	**Cluster-wise p-value**	**Talairach coordinates**		
				**x**	**y**	**z**
Lh Medial Orbitofrontal G	0.478	3738.51	0.00020	-4.3	43.1	-21.3
Lh Precentral G	0.468	2733.44	0.00330	-53.1	4.3	15.4
Rh Medial Orbitofrontal G	0.468	2207.54	0.01480	9.3	47.8	-7.1
**RBDSQ**						
**Cortical area**	**Correlation Coefficient**	**Cluster size (mm3)**	**Cluster-wise p-value**	**Talairach coordinates**		
				**x**	**y**	**z**
Rh Superior Frontal G	0.466	3880.97	0.00010	6.6	6.7	57.6
**Immediate recall (HLVT-R)**						
**Cortical area**	**Correlation Coefficient**	**Cluster size (mm3)**	**Cluster-wise p-value**	**Talairach coordinates**		
				**x**	**y**	**z**
Lh Superior Temporal G	0.453	2101.28	0.01920	-58.5	-32.2	9.7
Lh Caudal Middle Frontal G	0.419	5304.05	0.00010	-35.7	6.6	32.4
Rh Superior Frontal G	0.447	5757.86	0.00010	20.3	21.8	42.2
Rh Lateral Occipital G	0.449	1964.20	0.03050	30.3	-84.2	-6.9
**BJLO**						

**Cortical area**	**Correlation Coefficient**	**Cluster size (mm3)**	**Cluster-wise p-value**	**Talairach coordinates**		
				**x**	**x**	**x**
Lh Fusiform G	0.400	3126.78	0.00110	-31.1	1.2	-34.2
Rh Supramarginal G	0.390	2791.41	0.00350	56.7	-23.5	26.0

MDS-UPDRS III, Unified Parkinson's Disease Rating Scale III motor scores; UPSIT, University of Pennsylvania Smell Identification Test; RBDSQ, Rapid Eye Movement Behavior Disorder (RBD) Sleepiness Questionnaire; BJLO, Benton's Judgment of Line Orientation test; Lh, left hemisphere; Rh, right hemisphere; G, gyrus. All results were corrected for multiple comparisons using a cluster-wise threshold of $p < 0.05$ with Monte Carlo simulations. In addition they were also adjusted for age, sex and education.

Supplementary Table 3. Cortical thinning in iRBD patients that converted to a Lewy body disorder compared to non-converters

Converters vs Non-converters						
**Cortical area**	**Effect size (Cohen's d)**	**Cluster size (mm3)**	**Cluster-wise p-value**	**Talairach coordinates**		
				**x**	**y**	**z**
Lh Superior Frontal G	1.191	5674.91	0.00010	-7.1	46.1	38.1
Rh Precentral G	1.128	3632.43	0.00010	28.8	-9.2	45.5
Rh Lateral Occipital G	1.130	6479.15	0.00010	30.0	-79.7	11.9

Lh, left hemisphere; Rh, right hemisphere; G, gyrus. All results were corrected for multiple comparisons using a cluster-wise threshold of $p < 0.05$ with Monte Carlo simulations. In addition they were also adjusted for age, sex, education, baseline MDS-UPDRS III motor scores and time interval between baseline and last follow-up assessment.

Supplementary Table 4. Regions that showed cortical thinning in iRBD patients that converted to a Lewy body disorder compared to controls

Converters vs Controls					
Cortical area	Cluster size (mm3)	Cluster-wise p-value	Talairach coordinates		
			x	y	z
Lh Superior Frontal G	3010.06	0.00290	-8.2	18.6	50.1
Lh Inferior Temporal G	2931.15	0.00390	-41.3	-12.2	-26.6
Lh Superior Temporal G	8428.17	0.00010	-58.4	-37.6	14.7
Lh Lateral Occipital G	6865.43	0.00010	-41.3	-81.3	6.4
Rh Precentral G	9459.87	0.00010	48.3	-4.4	37
Rh Superior Temporal G	3739.04	0.00030	65.4	-30.0	9.8
Rh Lateral Occipital G	3871.06	0.00020	31.0	-78.9	12.1

Lh, left hemisphere; Rh, right hemisphere; G, gyrus. All results were corrected for multiple comparisons using a cluster-wise threshold of $p < 0.05$ with Monte Carlo simulations. In addition they were also adjusted for age, sex and education.

Supplementary Table 5. Subcortical volumes in converters, non-converters and controls

Subcortical area	Converters	Non-converters	CTR	Converters vs CTR (p value)	Non-converters vs CTR (p value)	Converters vs Non-converters (p value)
**Lh Thalamus**	6396.3 (818.3)	6964.3 (776.8)	7060.1 (864.4)	0.740	0.906	0.597
**Lh Caudate**	3150.7 (417.6)	3455.5 (789.0)	3376.3 (404.7)	0.799	0.970	0.884
**Lh Putamen**	4114.7 (735.4)	4692.6 (852.1)	4658.1 (560.3)	0.440	0.996	0.499
**Lh Pallidum**	1781.7 (312.1)	1961.4 (275.4)	1953.0 (222.4)	0.942	0.984	0.975
**Lh Accumbens**	394.0 (75.3)	420.3 (102.4)	446.1 (72.9)	0.622	0.286	0.176
**Lh Hippocampus**	3613.5 (470.0)	3814.0 (398.0)	3904.3 (371.4)	0.672	0.770	0.916
**Lh Amygdala**	1342.9 (194.5)	1520.8 (191.6)	1609.4 (141.4)	0.004	0.060	0.166
**Rh Thalamus**	6541.3 (1041.8)	7135.2 (910.2)	7119.3 (954.1)	0.747	0.976	0.685
**Rh Caudate**	3356.3 (440.2)	3540.3 (594.6)	3401.3 (392.9)	0.995	1.000	0.996
**Rh Putamen**	4026.3 (580.6)	4490.7 (772.1)	4549.4 (556.8)	0.658	0.984	0.570
**Rh Pallidum**	1784.0 (338.7)	1915.1 (266.4)	1834.6 (202.2)	0.958	0.988	0.982
**Rh Accumbens**	427.2 (54.5)	453.9 (91.3)	477.2 (58.4)	0.547	0.733	0.844
**Rh Hippocampus**	3703.3 (436.6)	3928.9 (396. 5)	4063.7 (409.5)	0.253	0.951	0.204
**Rh Amygdala**	1527.5 (290.7)	1713.4 (213.2)	1718.7 (137.6)	0.937	0.915	0.996

Lh, left hemisphere; Rh, right hemisphere; G, gyrus. All results were corrected for age, sex, education, TIV, and adjusted for multiple comparisons using FDR ($q > 0.05$).

Supplementary Table 6. Clinical characteristics of PD patients with and without probable RBD, iRBD and controls

	iRBD (n = 27)	CTR (n = 31)	PD without probable RBD (n = 94)	PD with probable RBD (n = 56)	PD without pRBD vs CTR (p value)	PD with pRBD vs CTR (p value)	PD without pRBD vs iRBD (p value)	PD with pRBD vs iRBD (p value)
**Age (mean, SD)**	68.9 (5.5)	58.5 (11.0)	59.3 (9.3)	62.9 (9.6)	0.684	0.058	**< 0.001**	**0.003**
**Sex (M/F)**	22/5	20/11	55/39	39/17	0.554	0.624	0.029	0.252
**Education (mean, SD)**	12.7 (5.2)	16.5 (3.1)	15.2 (9.2)	15.7 (2.8)	0.044	0.226	**0.002**	**0.001**
**MDS-UPDRS III (mean, SD, range)**	4.2 (3.6; 0-15)	0.32 (0.9; 0-4)	20.3 (9.2; 6-51)	21.6 (9.0; 6-41)	**< 0.001**	**< 0.001**	**< 0.001**	**< 0.001**
**Hoehn & Yahr (mean, range)**	0 (0-0)	0 (0-0)	1.55 (0.5; 1-3)	1.7 (0.5; 1-2)	**< 0.001**	**< 0.001**	**< 0.001**	**< 0.001**
**UPSIT (mean, SD, range)**	17.6 (6.2; 9-35)	36.7 (1.6; 34-40)	23.8 (8.1; 1-39)	19.0 (8.1; 5-37)	**< 0.001**	**< 0.001**	0.100	0.550
**RBDSQ (mean, SD, range)**	9.3 (2.9; 1-13)	2.1 (1.4; 0-4)	2.6 (1.2; 0-4)	9.3 (8.3; 5-46)	0.350	**< 0.001**	**< 0.001**	**0.002**
**ESS (mean, SD, range)**	8.4 (4.5; 0-20)	4.8 (3.1; 0-12)	5.1 (3.0; 0-13)	5.8 (3.5; 0-15)	0.654	0.361	**0.001**	**0.016**
**GDS (mean, SD, range)**	6.0 (2.1; 3-11)	5.2 (1.0; 2-7)	5.3 (1.5; 1-10)	5.3 (1.5; 2-11)	0.793	0.784	0.166	0.082
**MoCA (mean, SD, range)**	25.3 (4.5; 11-30)	28.3 (1.2; 27-30)	27.5 (2.1; 21-30)	27.0 (2.5; 19-30)	0.348	0.188	0.482	0.789
**Immediate recall (HVLT-R) (mean, SD, range)**	20.7 (5.4; 9-33)	26.7 (4.7; 16-35)	26.2 (5.1; 13-36)	23.5 (5.5; 11-35)	0.826	0.061	0.018	0.808

**Delayed recall (HVLT-R) (mean, SD, range)**	6.8 (3.0; 0-12)	10.0 (1.9; 6-12)	9.0 (2.5; 0-12)	7.7 (2.7; 0-12)	0.386	**0.001**	0.357	0.213
**Recognition (HVLT-R) (mean, SD, range)**	10.4 (1.5; 7-12)	11.7 (0.6; 10-12)	11.7 (1.8; 9-28)	11.0 (1.2; 8-12)	0.504	0.110	**0.008**	0.613
**Benton Judgment Line Orientation (mean, SD, range)**	11.5 (1.9; 8-15)	13.3 (1.8; 9-15)	13.2 (1.9; 8-15)	12.5 (2.1; 7-15)	0.459	0.190	**0.005**	0.365
**Letter and Number Sequencing (mean, SD, range)**	8.6 (3.1; 4-17)	11.9 (3.0; 8-20)	11.4 (2.6; 4-20)	10.2 (3.3; 2-20)	0.968	0.284	0.031	0.662
**Semantic fluency (mean, SD, range)**	44.3 (9.2; 27-65)	55.2 (9.5; 39-74)	52.9 (12.1; 20-103)	44.5 (10.5; 25-75)	0.317	**<0.001**	0.521	**0.011**
**Symbol and Digit Modalities Test (mean, SD, range)**	31.4 (9.3; 15-56)	49.0 (11.5; 30-76)	42.5 (10.2; 7-70)	40.1 (9.0; 16-60)	0.058	0.053	0.055	0.138

Values correspond to means followed by standard deviation or standard deviation and range. Comparisons between groups were performed using X^2, Mann-Whitney U tests, or ANOVA. Age and sex were included as covariates in the analyses of motor and non-motor variables, whereas education was included as an additional covariate in the analyses of cognitive variables. **Values in bold correspond to significant group differences after adjusting for multiple comparisons with false discovery rate corrections (FDR) (q < 0.05).**

Supplementary Table 7. Regions that showed cortical thinning in PD patients without RBD and with RBD compared to controls

PD without RBD vs Controls						
**Cortical area**	**Effect size (Cohen's d)**	**Cluster size (mm3)**	**Cluster-wise p-value**	**Talairach coordinates**		
				**x**	**y**	**z**
Lh Superior Frontal G	0.407	3705.30	0.00020	-6.7	-4.7	50.7
**PD with RBD vs Controls**						
**Cortical area**	**Effect size (Cohen's d)**	**Cluster size (mm3)**	**Cluster-wise p-value**	**Talairach coordinates**		
				**x**	**y**	**z**
Lh Superior Frontal G	0.737	4337.62	0.00020	-8.2	13.8	50.6
Lh Inferior Temporal G	0.508	4957.83	0.00010	-53.0	-25.5	-25.5
Lh Rostral Middle G	0.540	3025.51	0.00330	-41.2	29.0	27.5
Rh Inferior Temporal G	0.548	3168.08	0.00300	47.6	-18.4	-26.8
Rh Superior Frontal G	0.508	3705.84	0.00050	12.8	60.5	11.1

Lh, left hemisphere; Rh, right hemisphere; G, gyrus. All results were corrected for multiple comparisons using a cluster-wise threshold of $p < 0.05$ with Monte Carlo simulations. In addition they were also adjusted for age, sex and education.
